# The Structural Changes in the Membranes of *Staphylococcus aureus* Caused by Hydrolysable Tannins Witness Their Antibacterial Activity

**DOI:** 10.3390/membranes12111124

**Published:** 2022-11-10

**Authors:** Ewa Olchowik-Grabarek, Szymon Sękowski, Agnieszka Kwiatek, Jagoda Płaczkiewicz, Nodira Abdulladjanova, Vadim Shlyonsky, Izabela Swiecicka, Maria Zamaraeva

**Affiliations:** 1 Laboratory of Molecular Biophysics, Department of Microbiology and Biotechnology, Faculty of Biology, University of Bialystok, 15-245 Bialystok, Poland; 2 Department of Molecular Virology, Institute of Microbiology, Faculty of Biology, University of Warsaw, 02-096 Warsaw, Poland; 3 International Centre for Translational Eye Research, Institute of Physical Chemistry, Polish Academy of Sciences, 01-224 Warsaw, Poland; 4 Institute of Bioorganic Chemistry, Academy of Sciences of the Republic of Uzbekistan, Tashkent 100143, Uzbekistan; 5 Laboratory of Physiology and Pharmacology, Faculty of Medicine, Université libre de Bruxelles, 1070 Brussels, Belgium

**Keywords:** tannins, *Staphylococcus aureus*, bacterial membrane vesicles (MVs), antibacterial activity

## Abstract

Polyphenols, including tannins, are phytochemicals with pronounced antimicrobial properties. We studied the activity of two hydrolysable tannins, (i) gallotannin—1,2,3,4,5-penta-O-galloyl-β-D-glucose (PGG) and (ii) ellagitannin—1,2-di-O-galloyl-4,6-valoneoyl-β-D-glucose (dGVG), applied alone and in combination with antibiotics against *Staphylococcus aureus* strain 8324-4. We also evaluated the effect of these tannins on bacterial membrane integrity and fluidity and studied their interaction with membrane proteins and lipids. A correlation between the antimicrobial activity of the tannins and their membranotropic action depending on the tannin molecular structure has been demonstrated. We found that the antibacterial activity of PGG was stronger than dGVG, which can be associated with its larger flexibility, dipole moment, and hydrophobicity. In addition, we also noted the membrane effects of the tannins observed as an increase in the size of released bacterial membrane vesicles.

## 1. Introduction

The interaction of compounds with bacterial cell membranes plays a key role in the manifestation of their antimicrobial action. Such interaction can result either in the penetration of the compounds into the bacterial cell or in that their activities are exerted on the surface of bacteria. In both cases, a change in the physicochemical properties of the bacterial membrane occurs. The functional state of bacterial membranes is one of the most important factors in the survival/existence of bacteria. Even minor disturbances in the properties of membranes can lead to the disruption of barrier functions and cellular metabolism, manifested by the suppression of bacterial growth, the prevention of biofilm formation, the inhibition of production, and the release of toxins or the direct killing of bacterial cells [1,2,3].

Plant extracts and isolated phytochemicals have long attracted attention as effective antimicrobial agents. Despite the centuries-old history of use in traditional medicine, the mechanism of their action is not fully understood. Among plant compounds, polyphenols, including tannins, are of particular interest [4,5,6]. Especially hydrolysable tannins, which are polyesters of gallic (gallotannins) and hexahydroxydiphenic (ellagitannins) acids, have the greatest prospect of being used in medicine and veterinary practice due to their water-solubility and the lack of a negative effect on non-pathogenic bacteria [7,8]. The tannins can act as unabsorbable high-molecular compounds at the level of the gastrointestinal tract or as absorbable low-molecular compounds, with metabolites of their enzymatic cleavage acting on the whole organism [7,9].

In addition to direct antibacterial activity, tannins can have synergetic and antibiotic-modulating action, which, in an era of increasing resistance of bacteria to antibiotics, attracts important attention. Increasing the antibiotics’ effectiveness includes several mechanisms, such as the inhibition of enzymes that break down the antibiotics, efflux pump inactivation, and the disruption of the structural organization of bacterial membranes, contributing to the better penetration of the antibiotics into the cell [10].

*Staphylococcus aureus*, Gram-positive bacteria, is one of the strongest pathogens of the genus *Staphylococcus* and causes a wide range of diseases from mild skin infections to deadly diseases, e.g., pneumonia, meningitis, osteomyelitis, endocarditis, or sepsis. The intensity of the diseases induced by *S. aureus* depends primarily on the virulence of this species and the high ability to acquire resistance to antibiotics. *S. aureus* produces a plethora of virulence factors, including: (i) factors responsible for adhesion, invasion, and colonization, (ii) factors that inhibit the host immune response, and (iii) toxins (toxic agents) [11,12]. The distribution of *S. aureus* strains resistant to antibiotics is currently increasing, especially in community-acquired infections. Therefore, studies are being conducted to develop new non-toxic antimicrobial agents and alternative methods of combating bacteria among them based on combination therapy [8,10].

The antibacterial activity of plant extracts containing tannins or isolated compounds is well demonstrated on different strains of *S. aureus* [13,14,15,16,17,18]. However, the mechanism of this activity and the influence of the structure of compounds on the manifestation of this activity are poorly understood and the data are quite contradictory. For example, Puljula et al. [19] analyzed the activity of 22 ellagitannins and did not reveal a clear correlation between the number of free galloyl groups and the antibacterial activity against *S. aureus*. This observation allowed these authors to conclude that molecular flexibility did not play a significant role in the antimicrobial activity of ellagitannins. However, we have previously shown that the molecular weight of tannins and their flexibility determine their antimicrobial and antihemolytic activity against *S. aureus* [12].

Bearing this in mind, in the present work, we studied the antibacterial activity of selected tannins (gallotannin and ellagitannin) against *S. aureus* strain 8325-4 and their synergistic action with antibiotics. We also evaluated their effect on the bacterial membrane integrity, fluidity, and their interaction with membrane proteins and lipids and demonstrated a correlation between the antimicrobial activity of the tannins and their membranotropic action depending on the tannin molecular configuration. In addition, we found that the membrane effects of the tannins affected the formation of bacterial membrane vesicles.

## 2. Materials and Methods

### 2.1. Chemicals

Two hydrolysable tannins (gallotannin and ellagitannin), 1,2,3,4,5-penta-O-galloyl-β-D-glucose (PGG) and 1,2,-di-O-galloyl-4,6-valoneoyl-β-D-glucose (dGVG), were obtained and characterized as described previously [12]. Briefly, 1,6-diphenyl-1,3,5-hexatriene (DPH), 1-(4-trimethylammoniumphenyl)-6-phenyl-1,3,5-hexatriene (TMA-DPH), and antibiotics (methicillin, oxacillin, and ampicillin) were obtained from Merck (KGaA, Darmstadt, Germany). Fluorescent probes—Sytox Green Nucleic Acid Stain and FM4-64 were from Thermo Fisher Scientific (Walthman, Massachusetts, USA).

All microbiological media used in the study were supplied by Oxoid (Basingstoke, England). All other reagents were purchased from POCH (Gliwice, Poland).

### 2.2. Bacterial Strain and Growth Conditions

*S. aureus* strain 8325-4 obtained from Prof. Jan Oscarsson (University of Lund, Lund, Sweden) was used in the study. Bacteria were grown overnight at 37 °C in Mueller Hinton (MH) broth with shaking at 200 rpm or on nutrient agar (NA) plates.

### 2.3. Antimicrobial Activity

#### 2.3.1. Determination of Minimum Inhibitory Concentration (MIC) and Minimum Bactericidal Concentration (MBC)

A total of 180 μL of bacterial suspension (OD_600_ = 0.1) and 20 μL of the tested compounds (in the range of concentration of 19–76 μg/mL (20–80 μM) PGG and 38–286 μg/mL (40–300 μM) dGVG) were added to the wells of a sterile 96-well microtiter plate. Antibiotics were tested with different concentrations in the range of 0.0625–1.25 μg/mL. The control wells contained 20 μL of PBS. Optical density (OD_600_) was measured at 0, 2, 4, 6, 8, 10, and 12 h of incubation at 37 °C using a microplate reader SpectraMax M2 (Molecular Device, San Jose, USA). The lowest concentration of compound completely preventing the growth of bacteria was considered as its minimum inhibitory concentration (MIC).

Minimal bactericidal concentration (MBC) was assessed as follows. Five microliters of the overnight culture from each well in the plate with tested compounds of concentration equal to and higher than the MIC value were transferred into MH agar and incubated overnight at 37 °C. The MBC value was determined as the lowest concentration of compounds for which no bacterial growth on the nutrient agar was observed.

#### 2.3.2. Synergic Effects between PGG and β-Lactam Antibiotics against *S. aureus*

The interaction between the PGG and β-lactam antibiotics (methicillin, oxacillin, and ampicillin) against *S. aureus* was tested by the checkerboard method [20]. Bacterial cultures were grown in the presence of the following sub-inhibitory concentration of PGG: 1/2 MIC, 1/4 MIC, 1/8 MIC, and 1/16 MIC in combination with sub-inhibitory concentrations of antibiotics: 1/2 MIC, 1/4 MIC, 1/8 MIC, and 1/16 MIC. The MIC and MBC values are determined as in the section above. The synergistic effect was evaluated as the fractional inhibitory concentration (FIC) index. The FIC was calculated based on the following equations:

(1) $${\mathrm{FIC}}_{A}=\frac{{\mathrm{MIC}}_{A}\;\mathrm{in}\;\mathrm{combination}\;}{{\mathrm{MIC}}_{A}}$$

(2) $${\mathrm{FIC}}_{\mathrm{PGG}}=\frac{{\mathrm{MIC}}_{\mathrm{PGG}}\;\mathrm{in}\;\mathrm{combination}}{{\mathrm{MIC}}_{\mathrm{PGG}}}$$

(3) $${\mathrm{FIC}}_{\mathrm{index}}={\mathrm{FIC}}_{A}+{\mathrm{FIC}}_{\mathrm{PGG}}$$

The combination of antibiotic and PGG was defined as a synergistic effect if the FIC index is <0.5, a partial synergistic was defined as when the FIC index is between 0.5 and 1.0, and an additive effect if *FIC* is 1 [21].

### 2.4. Bacterial Cell Membrane Permeability Assay

The membrane permeability assay was carried out using Sytox Green Nucleic Acid Stain according to Roth et al. with modification [22]. Bacteria *S. aureus* was grown overnight at 37 °C in Mueller Hinton (MH) broth with shaking at 200 rpm. Next, the supernatant was removed by centrifugation at 2300× *g* for 15 min, and the bacterial cells were diluted in PBS with 5% MH broth. A total of 180 μL of bacterial suspension (OD_600_ = 0.01 in 10 mM PBS buffer, pH 7.4 with 5% MH broth) was added to each well of sterile 96-well black plates containing 20 μL of different final concentration tannins in the range of 1/4 MIC–3 MIC and incubated 1 h at 37 °C. The intensity of fluorescence was determined after 2 h incubation with 5 μM Sytox Green using the microplate reader SpectraMax M2 (Molecular Device, San Jose, USA). The measurements were carried out at the wavelengths λ_exc_. = 504 nm and λ_em_. = 523 nm. A bacterial suspension treated with 1% Triton X-100 was used as a positive control.

### 2.5. Measurements of S. aureus Membrane Fluidity

Bacteria of *S. aureus* were grown overnight at 37 °C in Mueller Hinton (MH) broth with shaking at 200 rpm. Next, the supernatant was removed by centrifugation at 2300× *g* for 15 min, and the bacterial cells were diluted in PBS. The bacterial suspension (OD_600_ = 0.01 in 10 mM PBS buffer, pH 7.4) was labeled with TMA-DPH at a final concentration of 1 μM (15 min, 37 °C). Then, the bacterial suspension was incubated with tannins in a concentration range of 2–20 μM (60 min, 37 °C). The fluorescence measurements were carried out at 37 °C using a PerkinElmer LS-55 (PerkinElmer, UK) spectrofluorometer equipped with a fluorescence polarization device. The readings were taken at intervals of 2 s. Changes in membrane fluidity after the addition of tannins were determined based on the anisotropy values of the samples (r). The anisotropy values (r) were calculated by the fluorescence data manager program using the Jablonski equation:

(4) $$r=\frac{I_{\mathrm{VV}}-{\mathrm{GI}}_{\mathrm{VH}}}{I_{\mathrm{VV}}+2{\mathrm{GI}}_{\mathrm{VH}}}$$

where I_VV_ and I_VH_ are the vertical and horizontal fluorescence intensities, respectively, of the vertical polarization of the excitation light beam. The factor G = I_HV_/I_HH_ (grating correction factor) corrects the polarizing effects of the monochromator. The excitation wavelength was 340 nm, and the fluorescence emission was measured at 430 nm for TMA-DPH [23]. Based on the data obtained, the membrane-ordering parameter was calculated using the following equation [12]:

(5) $$S=\frac{\sqrt{\left[1-2\left(\frac{r}{r_{0}}\right)+5{\left(\frac{r}{r_{0}}\right)}^{2}\right]}-1+\frac{r}{r_{0}}}{2\left(\frac{r}{r_{0}}\right)}$$

where r_0_ is the fluorescence anisotropy of TMA-DPH in the absence of any rotational motion of the probe. The theoretical value of the r_0_ of TMA-DPH is 0.4.

### 2.6. Detection of Tannin-Bacterial Membrane Interaction by Measuring DPH Quenching of Fluorescence

Bacteria of *S. aureus* were grown overnight at 37 °C in Mueller Hinton (MH) broth with shaking at 200 rpm. Next, the supernatant was removed by centrifugation at 2300× *g* for 15 min and bacterial cells were diluted in PBS. The bacterial suspension (OD_600_ = 0.01 in 10 mM PBS buffer, pH 7.4) was labeled with DPH at a final concentration of 1 μM (15 min, 37 °C). Then, the bacterial suspension was incubated with tannins in a concentration range of 2–20 μM (60 min, 37 °C). The DPH fluorescence intensity was registered at λ_exc_. = 348 nm and λ_em_. = 426 nm. The tannin incorporation into bacterial membranes was analyzed by measuring the fluorescence quenching of DPH-stained *S. aureus* [24].

In order to evaluate the potency of tannins to build into bacteria cell membranes, the Stern–Volmer constants that characterize the affinity of the used compounds to membranes stained by DPH were calculated based on the following equation [25]:

(6) $$\frac{F_{0}}{F}=1+{\;K}_{\mathrm{SV}}\;\left[Q\right]$$

where: F_0_ and F are DPH fluorescence without and in the presence of PGG/dGVG; K_SV_ is the Stern–Volmer constant; and [Q] is the concentration of the quencher (PGG or dGVG, respectively).

### 2.7. Fluorescence Analysis of Membrane Proteins of S. aureus—Tannin Interaction

Bacteria of *S. aureus* were grown overnight at 37 °C in Mueller Hinton (MH) broth with shaking at 200 rpm. Next, the supernatant was removed by centrifugation at 2300× *g* for 15 min, and the bacterial cells were diluted in PBS. A total of 990 μL of bacterial suspension (OD_600_ = 0.1 in 10 mM PBS buffer, pH 7.4) with 10 μL of tannins were incubated for 60 min at 37 °C. In total, 10 μL of water (PGG and dGVG solvent) was added to the control. The fluorescence spectra of bacteria membrane proteins without and with tannins were registered in the range of 320–420 nm. The excitation wavelength was set at 295 nm. The scanning speed was 60 nm/min. The fluorescence spectra of the membrane proteins of *S. aureus* (without and with tannins) were corrected for the proper baselines. The analyses were carried out using a PerkinElmer LS-55B (PerkinElmer, UK) spectrofluorimeter. Based on the fluorescence spectra, the single values of fluorescence descended from the Trp residues (λ_exc_. = 295 nm and λ_em_. = 350 nm) were read in order to calculate the Stern–Volmer constants.

To determine the mechanism of fluorescence quenching that occurred (static or dynamic), quenching constant (k_q_) values have been calculated based on the below equation [25]:

(7) $$k_{q}=\frac{K_{\mathrm{SV}}}{\tau_{0}}$$

where: k_q_—quenching constant; K_SV_—Stern–Volmer constant; τ_0_—fluorescence lifetime of fluorophore molecules (5 × 10^−9^ s).

### 2.8. Isolation of Naturally Secreted MVs

Membrane vesicles (MVs) secreted from *S. aureus* were isolated according to the method described by Prados-Rosales et al. with minor modifications [26]. Prior to the isolation of naturally secreted MVs, *S. aureus* was cultivated with 40 μM PGG, 40 μM dGVG, and in the control conditions for 12 h at 37 °C. Then, the supernatants obtained from the *S. aureus* liquid cultures were passed through the 0.22 μm filter units (Roth Chemicals, Zielona Góra, Poland), and to prevent proteolysis, EDTA-free Complete Protease inhibitor mixture tablets (Roche, Warsaw, Poland) were added. Subsequently, crude MVs were purified by ultracentrifugation at 32,000× *g*, 12 h, 4 °C in the step gradient (25–40%) of OptiPrep™ Density Gradient MediumMerck (KGaA, Darmstadt, Germany) at the Beckman Coulter ultracentrifuge using an SW 32 Ti swinging-bucket rotor. The purified MVs were visualized by Transmission Electron Microscopy, as follows: MVs were adsorbed on a formvar/carbon-coated copper grid for 5 min and stained with 2% ammonium molybdate for 1 min. The concentration of the obtained MVs was calculated on the basis of protein and lipid content by the colorimetric method using a Pierce Modified Lowry Protein Assay Kit (Thermo Fisher Scientific, Walthman, MA, USA) and by an Infinite 200 PRO Tecan plate reader (Tecan, Switzerland) using the lipophilic dye FM4-64 (Thermo Fisher Scientific, Walthman, MA, USA) that fluoresces upon incorporation into a lipid environment.

### 2.9. Statistical Analysis

The results are presented as mean ± SD. The level of significance was analyzed using a one-way ANOVA test. *p* < 0.05 and below was accepted as statistically significant. Statistical analysis was performed using Origin 8.5.1 software (Microcal Software Inc., Northampton, MA, USA).

## 3. Results and Discussion

### 3.1. Antibacterial Activities of the Studied Tannins, Applied Alone and in Combination with Antibiotics

A significant increase in bacterial resistance to antibiotics and the transfer of genes encoding antibiotic resistance from animal strains to human ones were previously observed on a global scale. Therefore, studies are being conducted to search for novel antibiotics and to develop alternative methods of combating bacteria such as combination therapy. The use of combinations of antibiotics and natural substances with antimicrobial properties may be an effective strategy. From these perspectives, polyphenols may increase the activity of traditional antibiotics used against a wide set of bacteria [27,28,29,30].

The antibacterial activity of two hydrolysable tannins, (i) gallotannin 1,2,3,4,5-penta-*O*-galloyl-β-D-glucose (PGG) and (ii) ellagitannin 1,2-di-*O*-galloyl-4,6-valoneoyl-β-D-glucose (dGVG), against the Gram-positive *S. aureus* strain 8325-4 was investigated. The studied compounds possess similar molecular weights and have the same gallic acid residues (5) and hydroxyl groups (15). However, they differ in the degree of flexibility of the molecule. This is due to the absence (PGG) or presence (dGVG) of a valoneoyl group (gallic acid trimer), which limits the mobility of the gallic acid residues in the molecule and causes its higher rigidity [24].

**Figure 1:**
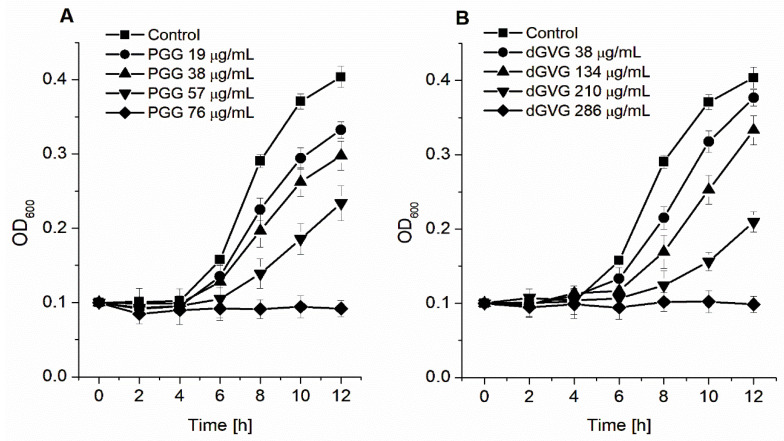
Growth curves of *S. aureus* 8325-4 in the presence of PGG (**A**) and dGVG (**B**). The data presented are the means ± SD, n = 6.

First, we determined the minimum inhibitory concentration (MIC) and the minimum bactericidal concentration (MBC). Figure 1 shows that both tannins tested modified the *S. aureus* growth in a concentration-dependent manner.

However, the complete inhibition of bacterial growth was obtained at significantly different tannin concentrations. While for PGG, the MIC value was 76 μg/mL, for dGVG, it reached 286 μg/mL (Table 1). The MIC for PGG was 3.75-fold lower compared to dGVG, indicating a much stronger antimicrobial effect of PGG against *S. aureus*. The bactericidal activity of the tannins was consistent with their bacteriostatic effect. In these studies, PGG also had stronger activity in comparison with dGVG. The MBC value determined for PGG was 304 μg/mL, whereas that for dGVG was much higher than 953 μg/mL (Table 1). In our previous research [12], we observed similar differences in the protective effects between PGG and dGVG on hemolysis induced by two *S. aureus* strains (8325-4 and NCTC 5655) and isolated α-toxin.

In the following experiments, we also checked whether PGG as a polyphenolic compound with marked antibacterial properties could contribute to an increase in the activity of such β-lactam antibiotics as methicillin, oxacillin, and ampicillin. The experiment was performed by a checkerboard method using a mixture of the antibiotic with PGG in various combinations, at concentrations below the determined MIC values of 1/2 to 1/16 (Table 1) (sub-MIC) obtained for both the components individually.

The MICs and MBCs for methicillin, oxacillin, and ampicillin against *S. aureus* strain 8325-4 were determined in an identical manner as those for the tannins, and the results are listed in Table 2.

**Figure 2:**
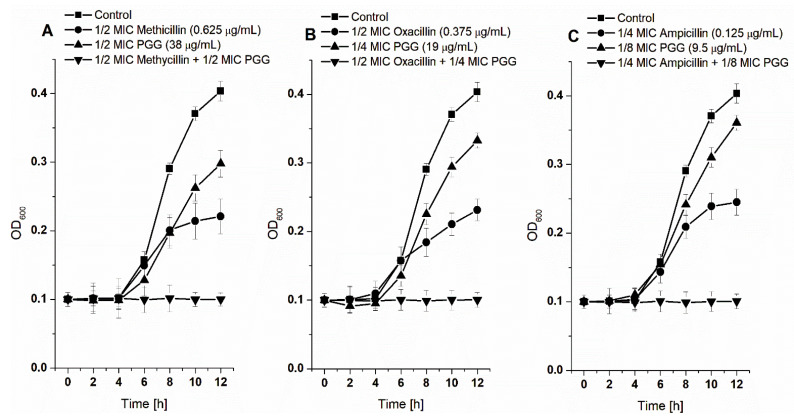
Growth curves of *S. aureus* 8325-4. Bacteria were grown at sub-inhibitory concentrations of PGG alone, antibiotic alone (methicillin (**A**), oxacillin (**B**), ampicillin (**C**)), and in combination. The data presented are the means ± SD, n = 6.

As shown in Figure 2, the presence of PGG in the mixture with antibiotics at sub-MIC concentrations increases their activity against *S. aureus* 8235-4 compared to their individual use at the same concentrations. To determine the nature/type of the interaction of PGG with antibiotics, we calculated the values for the FIC index according to Equations (1)–(3) given in the methods section. It is known that an interaction is considered synergistic if the FIC is <0.5, partial synergistic if the FIC is between 0.5 and 1.0, and additive if the FIC is 1.0 [21]. Different types of interaction were obtained depending on the antibiotic used (Table 2).

PGG in combination with ampicillin showed a synergistic effect, as evidenced by the FIC value that was 0.375. In this system, the MIC obtained for ampicillin was decreased four-fold in the presence of PGG at a sub-inhibitory concentration (1/8 MIC). For the PGG–oxacillin combination, the FIC value was 0.75. This indicates a partial synergistic effect. In this case, the MIC of oxacillin decreased two-fold, with a four-fold reduction in the PGG concentration. The combination of PGG with methicillin reduced the concentration, causing the inhibition of bacterial growth, but the effect achieved was additive rather than synergistic (FIC = 1). Similar results were obtained by Kirmusaoglu [21], who studied the activity of β-lactam antibiotics in combination with tannic acid against five *S. aureus* strains. The tannic acid in combination with ampicillin showed a synergistic effect against all the tested strains, reducing the MIC value of ampicillin by 2–16 times. In the same study, the combination of tannic acid with oxacillin showed partially synergistic or synergistic effects depending on the type of *S. aureus* strain. In contrast, Basri et al. [31] showed that tannic acid in combination with oxacillin exerted an additive effect against Vancomycin-intermediate *S. aureus* (VISA). This demonstrates that the type of tannin–antibiotic interaction as well as the final effect (synergistic or additive) were dependent on both the types of antibiotic/compound and the bacteria strain.

Furthermore, the combination of PGG with antibiotics at sub-MBC concentrations increased their bactericidal activity (Table 3). The obtained MBC values were decreased two-fold for the PGG-methicillin and PGG-oxacillin combinations. The strongest activity was observed with the PGG-ampicillin combination, where the MBC values were decreased four-fold relative to these compounds used alone.

In comparison with dGVG, the stronger antibacterial activity of PGG is probably a result of the differences in the physicochemical parameters of its molecules. As we demonstrated in our previous study [24], the more flexible PGG molecules have a smaller surface and volume but higher dipole moment and hydrophobicity compared to dGVG. Therefore, we suggest that these features of PGG impart them greater ability to interact with bacteria membranes, making PGG a more efficient antibacterial compound in comparison with dGVG. To verify this hypothesis, we further studied the interaction of tannins with *S. aureus* bacterial membranes.

### 3.2. Influence of PGG and dGVG on the S. aureus Cell Membrane—Fluorescence Studies

It is well known that bacterial cell membrane functioning takes an important role in many physiological processes, e.g., the respiration process, osmoregulation, biosynthesis, or transport processes [32]. Therefore, the modification and/or disturbances of bacterial membrane biophysical and biochemical properties can result in bacteria metabolic stress and dysfunction, finally leading to cell death. Hence, the antimicrobial activity of the tested tannins against *S. aureus* demonstrated above may be due to the interactions of tannins with the bacterial membrane, which lead to the modification of the membrane structure, resulting in changes in the function of the bacterial cell. Previously, a correlation was shown between antimicrobial action and the damage of bacterial membranes for a number of polyphenols like resveratrol-derived [33], catechins [34], as well as condensed and hydrolysable tannins [35].

**Figure 3:**
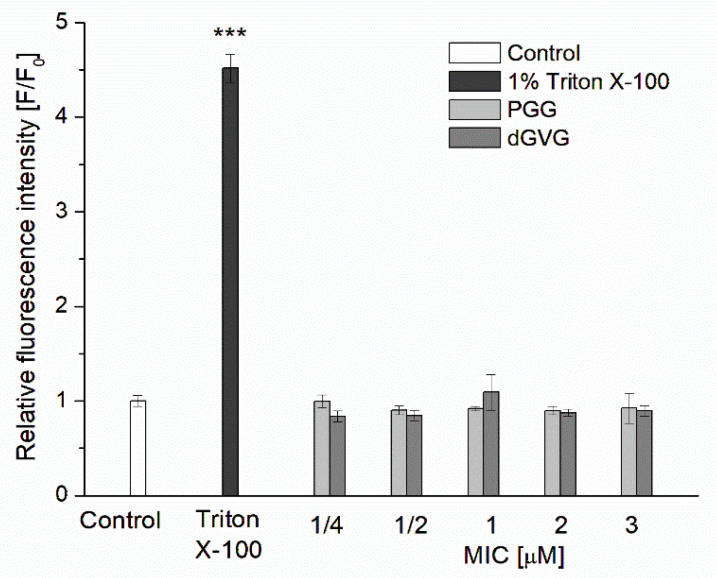
Influence of PGG and dGVG on *S. aureus* cell membrane integrity. Briefly, 1% Triton X-100 was used as a positive control. Bacterial cells labeled with Sytox Green in the presence of PGG and dGVG in the concentration range of ¼ x MIC to 3 x MIC, which corresponds to 20 μM, 40 μM, 80 μM, 160 μM, and 240 μM for PGG and 75 μM, 150 μM, 300 μM, 600 μM, and 900 μM for dGVG. The data presented are the means ± SD, n = 6. Statistical significance was estimated using a one-way ANOVA test (results compared to control, *** *p* < 0.001).

We first analyzed the effect of tannins on the membrane integrity of *S. aureus* using Sytox Green, which penetrates damaged membranes and shows fluorescence when bound to the nucleic acids of the bacterial cell. As shown in Figure 3, bacterial cells labeled with Sytox Green in the presence of PGG and dGVG showed no statistically significant increase in fluorescence intensity compared to the control after 1 h of incubation. The same effect was still observed even after 12 h of incubation. The pre-incubation of bacteria with 1% Triton X-100 used as a positive control caused a statistically significant 4.5 times increase in the fluorescence intensity of the Sytox Green probe that indicates the damage to the bacterial membrane and is consistent with the literature [36]. No effect on the integrity of *S. aureus* bacteria has been shown for epigallocatechin gallate (EGCG)—ester catechin with gallic acid. This compound at the MIC concentration did not induce damage to *S. aureus* membranes and at lower concentrations even inhibited the Sytox Green uptake induced by antibacterial peptide nisin [37].

**Figure 4:**
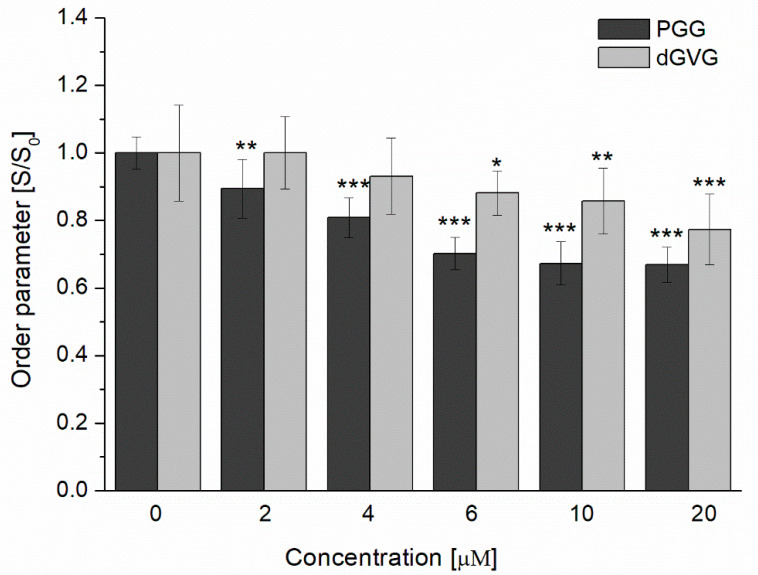
Changes in the order parameter S/S_0_ of the *S. aureus* membrane measured using a TMA-DPH probe in the presence of PGG and dGVG. The data presented are the means ± SD, n = 6. Statistical significance was estimated using a one-way ANOVA test (results compared to control, * *p* < 0.05; ** *p* < 0.01; *** *p* < 0.001).

In an attempt to further elucidate the mechanism of the antibacterial action of the studied tannins, we studied next their effect on the structure of membranes. The tannins’ influence on the structure of bacteria cell membranes was investigated using fluorescence techniques by applying such dyes as TMA-DPH and DPH located at different parts of the phospholipid bilayer [24]. TMA-DPH anchors in the outer, polar parts of the membrane at the C4 level, whereas DPH enters into the deeper, hydrophobic region of phospholipids. In the study, the changes in fluorescence anisotropy were monitored, and the lipid order parameter (S) has been calculated using Equation (5) based on the obtained data. The results are presented as the ratio (S/S_0_), where S_0_ and S represent lipid order parameters in the absence and in the presence of PGG and dGVG. Figure 4 demonstrates that PGG in the concentration range of 2–20 µM changed more pronouncedly the lipid order parameters in comparison with dGVG. Therefore, it can be concluded that PGG interacts more strongly than dGVG with the staphylococcal membrane. For the highest concentration (20 µM), the lipid order parameter of the polar parts of *S. aureus* membranes changed from 1 (as control) down to 0.668 ± 0.052 and 0.773 ± 0.105 for PGG and dGVG, respectively, which means that hydrophilic zone of the lipid bilayer became more fluid in the presence of both PGG and dGVG.

**Figure 5:**
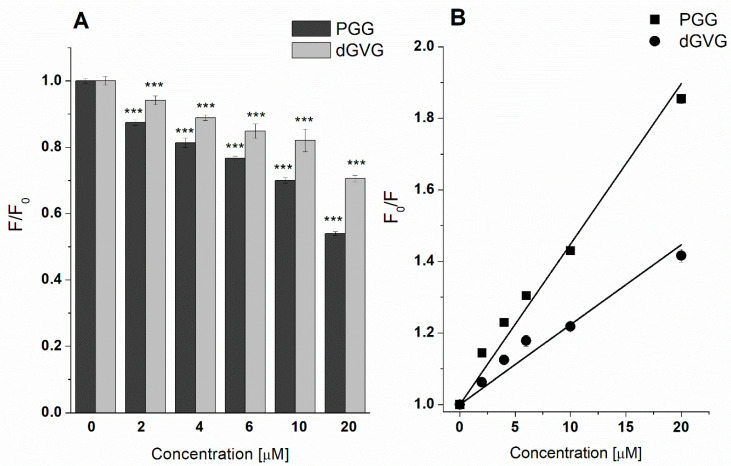
Fluorescence decrease (**A**) and Stern–Volmer plots (**B**) of *S. aureus* membrane stained with DPH. Values presented as relative fluorescence. The data presented are the means ± SD, n = 6. Statistical significance was estimated using a one-way ANOVA test (results compared to control, *** *p* < 0.001).

In order to better characterize the physicochemical interaction of PGG and dGVG with the *S. aureus* membrane, bacteria cells have been stained by DPH, and the fluorescence quenching of this label has been analyzed. In the presence of the studied compounds, a fluorescence decrease has been observed in a concentration-depended manner (Figure 5A). Even the lowest concentration of PGG and dGVG (2 µM) was able to decrease the DPH fluorescence, which confirmed the interaction of these compounds with the non-polar parts of the bacteria membrane. At the highest concentration of the studied tannins, we observed a decrease from 1 (control) to 0.54 ± 0.01 (for PGG) and 0.71 ± 0.01 (for dGVG). Accordingly, it can be concluded that PGG more efficiently decreases DPH fluorescence in comparison with dGVG, which means a stronger interaction with bacteria membrane lipids as well as a greater ability to enter into the non-polar parts of the membrane. Based on the fluorescence measurements, binding constants were calculated in order to illustrate the differences in the PGG and dGVG influence on the *S. aureus* bacteria membrane. First, the Stern–Volmer constants, which characterize the affinity of the used compounds to membranes stained by DPH, were calculated based on Equation (6) [25] and Stern–Volmer plots (Figure 5B).

The obtained K_SV_ for PGG was two times larger in comparison with dGVG ((4.39 ± 0.09) × 10^4^ M^−1^ vs. (2.19 ± 0.12) × 10^4^ M^−1^). These results clearly demonstrate that both tannins have the ability to interact with the hydrophobic parts of staphylococcal cell membranes. Stronger activity was observed for PGG, and it was probably directly related to the molecular structure as well as to the higher hydrophobicity of the PGG molecules that favored better interactions with *S. aureus* cells in comparison to dGVG. Similar results were observed on liposomes and described in our previous work [24]. The important role of the hydrophobicity of the compounds in the manifestation of antimicrobial activity has been shown for flavonoids [38].

### 3.3. Evaluation of Interaction of Tannins with Membrane Proteins by Tryptophan Fluorescence

Among all the known polyphenols, tannins are characterized by a high affinity for proteins, and their antimicrobial activity can also be due to the formation of complexes with membrane proteins [9,39,40]. The interaction of tannins with proteins occurs mainly through non-covalent bonds, e.g., hydrophobic, van der Waals, electrostatic, and hydrogen bonds. This interaction can lead to the modification or inactivation of the functional activity of bacteria. For example, the interaction of polyphenols with membrane proteins has been shown to induce morphological changes and damage to the membrane integrity of *S. aureus* [41] and also to disrupt the adhesion process [40]. One of the basic methods for studying the interaction of the compounds with proteins is to evaluate the change in the fluorescence of aromatic amino acids (tryptophan, tyrosine, and phenylalanine). Tryptophan fluorescence is particularly investigated because it is most sensitive to changes in the chromophore microenvironment. In this regard, we studied the effect of tannins on the fluorescence of tryptophan residues of *S. aureus* membrane proteins as an indicator of their interaction with proteins.

**Figure 6:**
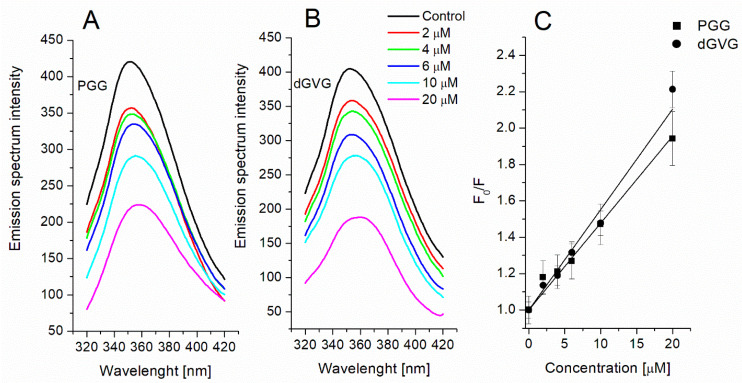
Fluorescence spectra of *S. aureus* membrane proteins without and in the presence of PGG (**A**) and dGVG (**B**) with the Stern–Volmer plots of tryptophan quenching (**C**). The data presented are the means ± SD, n = 6.

Figure 6A,B shows a fluorescence spectrum of membrane proteins at the excitation of 295 nm with a peak at 350 nm, characteristic of tryptophan. The incubation of bacterial cells with tannins resulted in strong fluorescence quenching in a concentration-dependent manner. At the highest tannins concentrations (20 µM), the fluorescence decreased to 51.5 ± 4.3% for PGG and 45.1 ± 2.8 for dGVG, respectively. The analysis of the fluorescence spectra also showed a concentration-dependent red-shift in the emission maxima of the spectra. At the 20 µM concentration of the tannins, the red-shifts for PGG and dGVG were 8 and 10 nm, respectively. These data suggest a movement of tryptophan residues into the hydrophilic environment or a change in the tryptophan environment to a more hydrophilic one. It should be mentioned that red-shift in the spectra was also noticed when studying the interaction of a coumarinic acid derivative with the membrane proteins of *S. aureus* [41].

For a more complete characterization of the interaction of tannins with membrane proteins, the obtained fluorescence quenching data were described by a Stern–Volmer equation (Equation (6), and its graphic form was represented as a Stern–Volmer plot (Figure 6C). Based on the Stern–Volmer plot, the Stern–Volmer constants were calculated and listed in Table 4.

The calculated K_SV_ value for dGVG is only insignificantly higher in comparison with the K_SV_ obtained for PGG. It is known that fluorescence quenching occurs via two types of mechanisms, static and dynamic mechanisms. Static quenching occurs when the quencher and the fluorescent molecule come into direct strong contact with the formation of a complex between them. In this case, the excitation energy of the fluorophore is transferred to the molecule of the quencher, followed by its dissipation in the form of heat. The dynamic mechanism involves only a collision between the molecules of the fluorophore and the quencher, which leads to an increase in their lifetime and facilitates vibrational relaxation [25].

To elucidate the mechanism of interaction of the tannins with proteins of such a complex system as the membrane, we calculated bimolecular quenching constants “k_q_” using Equation (7). The k_q_’s values obtained (Table 4) were greater than the value for the maximum scatter collision constant (2 × 10^10^ M^−1^s^−1^), which indicates the static quenching mechanism (complex formation as opposed to dynamic collisions) and it is in line with our data and those obtained by other authors when studying the interaction of tannins with isolated proteins [42,43].

In general, our findings indicate a strong interaction between the tannins and membrane proteins, which might cause a disorder in the metabolism of bacterial cells and, as a result, the bacteriostatic and bactericidal effects of the tannins described above. It was shown earlier that polyphenols are capable of inhibiting such vital protein systems in *S. aureus* as the membrane efflux system [44], ATP synthase [38], and antioxidant defense enzymes as catalase and SOD [45]. The slightly stronger activity of dGVG towards proteins at high concentrations than that of PGG is apparently due to the different structures of the compounds and their localization in bacterial membranes. PGG, being a more hydrophobic compound, penetrates deeper into the membrane and causes stronger changes in the lipid part of the membranes, while dGVG is localized more on the surface (DPH fluorescence quenching data, Figure 5) and thus has a greater ability to interact with membrane surface proteins. However, the overall bacterial effect of a particular tannin is the sum of its interactions with both bacterial proteins and lipids. PGG has a much greater effect on lipids than dGVG, which probably determines its strongest antibacterial effect as shown by its MIC and MBC values.

### 3.4. The Influence Tannins on Characteristics of Membrane Vesicles (MVs) Isolated from S. aureus

Membrane vesicles (MVs) released by Gram-positive bacteria play crucial role in bacterial survival in response to stress factors and participate in bacterial communication and host–pathogen interactions. Although they have many biological functions, the knowledge of their biogenesis mechanisms remains unclear [46,47,48,49,50].

**Figure 7:**
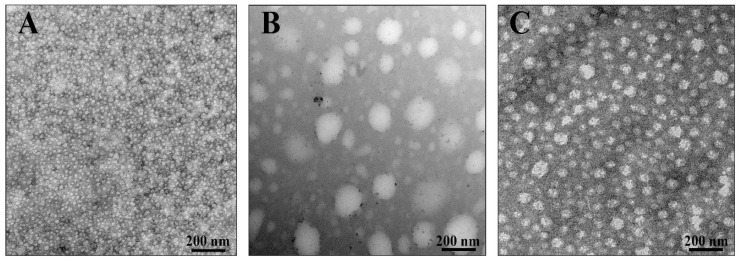
Images of purified *S. aureus* membrane vesicles visualized by transmission electron microscopy. (**A**) MVs released by bacteria cultivated without tannins, (**B**) cultivated with PGG 40 µM, (**C**) cultivated with dGVG 40 µM. *S. aureus* MVs were purified by ultrafiltration and ultracentrifugation. Images are representative of at least three independent experiments, and representative images are shown. Scale Bar: 200 nm.

As we demonstrated above, the studied compounds interact with the proteins and lipids of *S. aureus*; this may cause a change in the physicochemical properties of the membrane. We suggested that changes in the membrane properties could lead to the shape, size, and biopolymer composition modifications of MVs that are released by *S. aureus*. Therefore, we assessed the differences in the morphology and composition of the membrane vesicles (MVs) produced by *S. aureus* in the presence of PGG and dGVG. In order to visualize the MVs’ structure in the absence and in the presence of the studied compounds, transmission electron microscopy (TEM) has been applied (Figure 7).

**Figure 8:**
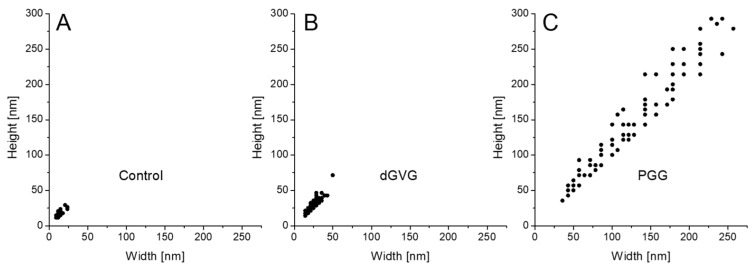
The size distribution of purified *S. aureus* membrane vesicles. MVs were released by bacteria cultivated in the absence of the tannins of control (**A**), 40 µM dGVG (**B**), and 40 µM PGG (**C**). n = 100 for each condition.

As demonstrated in Figure 8, the vesicles produced by *S. aureus* in the absence of tannins have oval dimensions with a width of 8.8–23.5 nm, a height of 11.8–26.5 nm, and a height/width ratio of 1.214 ± 0.218 (n = 100). In the presence of dGVG, the size of the vesicles increased to 14.28–49.9 nm × 14.28–71.4 nm (width × height) without a significant concomitant change in the ovalness (height/width ratio = 1.168 ± 0.160, n = 100, *p* = 0.09 vs. control). PGG induced dramatic change in the size distribution with a width in the range of 35.7–242.8 nm and a height in the range of 35.7–292.7 nm. The height/width ratio for these vesicles was 1.151 ± 0.143, indicating a significant modification of their spatial dimensions compared to the control (*p* = 0.038 vs. control, n = 100). Since the major mechanism of vesicle formation is a “bubbling” via holes in the proteoglycan layer on the membrane of *S. aureus* [51], and as the bacterial membrane becomes more fluid under tannin action (Figure 4), one can assume that bubbling may produce larger vesicles under such conditions, and their spatial dimensions can closer approach a spherical form for the same reasons. PGG as a more hydrophobic compound is more deeply embedded in a lipid palisade (Figure 5) and causes a stronger increase in the fluidity of bacterial membranes (Figure 4) and as a consequence more significant changes in vesicle sizes.

**Figure 9:**
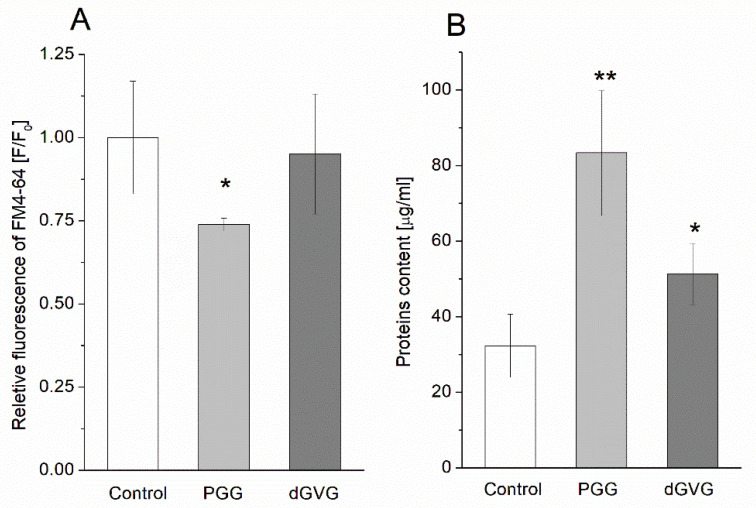
(**A**) Lipid and (**B**) protein quantifications of purified *S. aureus* membrane vesicles released by bacteria cultivated without tannins, with PGG 40 µM, and with dGVG 40 µM. Lipid quantification was determined by fluorescent FM4-64 dye and is presented as the ratio of the fluorescence in the sample with tannins (F) to the fluorescence of the control sample (Fo). Protein concentration was measured by Lowry Assay. Data are represented as mean ± SD of three biological replicates. * *p* < 0.05, ** *p* < 0.01.

We also evaluated the changes in the content of lipids and proteins under the influence of PGG and dGVG in isolated staphylococcal MVs. The results are demonstrated in Figure 9. It was observed that the relative quantity of lipids was reduced in the presence of PGG to 0.74 ± 0.02 in comparison to the control (1.00 ± 0.17) in a statistically significant manner (*p* = 0.029). For dGVG, only a slight and statistically insignificant decrease in lipids content was observed. The quantity of proteins in the vesicles isolated from bacteria increased to 83.34 ± 16.51 μg/mL (*p* = 0.008) and 51.34 ± 8.08 μg/mL (*p* = 0.037) for PGG and dGVG, respectively, if compared to the control (32.34 ± 8.33 μg/mL). In this case, there is also a clear correlation between the molecular structure of tannins, their membrane effects, and the influence on the vacuolization process and the composition of vesicles. PGG caused the strongest changes in the lipid and protein content of the vesicles.

Our studies demonstrated for the first time that tannins possess the ability to change the size as well as the chemical composition of membrane vesicles (MVs) released by Gram-positive bacteria. The obtained data are in accordance with the literature. It is known that antibacterial compounds are antimicrobial stressors encountered by bacteria, and environmental stresses are one of the inducers of vesiculation in bacteria. Under stress conditions, microorganisms can change their vesiculation pattern as a protective response causing bacterial membrane vesicles to feature diverse characteristics. Such secreted membrane vesicles can differ in lipid and protein composition or include misfolded proteins, which can be exported to the outside of the cell [52,53]. However, for a more complete characterization of this phenomenon, further studies of protein and lipid profiles are necessary because they determine the structure and functional activity of MVs. It cannot also be excluded that the increase in the activity of antibiotics by the studied tannins is also associated with a distribution of the MVs biogenesis as they are known to play a significant role in resistance of bacteria to antibiotics [51].

## 4. Conclusions

In this study, we have shown that the tannins PGG and dGVG, which have the same number of functional OH groups (15) and five aromatic rings but differ in molecular structure and spatial orientation, introduce structural changes of varying degrees in the bacterial membranes of *S. aureus* that in turn increase fluidity. The PGG effect was significantly stronger than apparently can be associated with greater flexibility due to the absence of the valoneoyl group and the free rotation of gallic acid residues, a smaller surface area, and the hydrophobicity of PGG [24], whereas dGVG is rather stiff in comparison with PGG due to the presence of the valoneoyl group in the molecule, which limits its spatial reorganization and the ability to interact with the lipid backbone of the membrane, as shown by experiments with DPH (Figure 5). The results obtained are in good agreement with the MIC and MBC values that were much lower for PGG and indicate the importance of the flexibility and the rotational mobility of gallic acid residues of tannins in exerting antimicrobial activity. Although the changes noticed in the structures of bacterial membranes did not lead to a disturbance in the integrity of the bacterial membranes, it is, however, possible to assume that these changes are also responsible for the enhancement of the antimicrobial activity of the antibiotics studied. Previously, it was shown that one of the mechanisms of enhancing the action of lactam antibiotics by galloylcatechins is their ability to modify the fluidity of *S. aureus* membranes [54]. Moreover, the structural changes in the bacterial membrane caused by PGG lead to disruption in the production of virulent membrane vesicles in *S. aureus*. Since MVs are crucial for bacterial survival, PGG as a compound leading to the modification of the MVs’ structure and content can, therefore, be potentially useful to disturb and hinder bacterial physiological processes connected with MV secretion.

## Figures and Tables

**Table 1:** Antibacterial activity (MIC and MBC) of PGG, dGVG, and β-lactam antibiotics against *S. aureus* 8325-4.

Compound	MIC [μg/mL]	MBC [μg/mL]
PGG	76 (80 μM)	304 (320 μM)
dGVG	286 (300 μM)	>953 (1000 μM)
Methicillin	1.25	5
Oxacillin	0.75	3
Ampicillin	0.5	2

**Table 2:** Antibacterial effect of PGG combined with antibiotics (MIC).

Combination	FIC Value	Effect	Range of Combination MICs [μg/mL]	Reduction Fold of MICs
PGG-Methicillin	1	Additive	38–0.625	2–2
PGG-Oxacillin	0.75	Partial synergism	19–0.375	4–2
PGG-Ampicillin	0.375	Synergism	9.5–0.125	8–4

**Table 3:** Antibacterial effect (MBC) of PGG combined with antibiotics.

Combination	Range of Combination MBCs [μg/mL]	Reduction Fold of MBCs
PGG–Methicillin	152–2.5	2–2
PGG–Oxacillin	152–1.5	2–2
PGG–Ampicillin	76–0.5	4–4

**Table 4:** Calculated Stern–Volmer (Ksv) and bimolecular quenching (k_q_) constants for PGG and dGVG interaction with *S. aureus* membrane proteins.

	PGG	dGVG
K_sv_ [M^−1^]	(4.74 ± 0.94) × 10^4^	(5.75 ± 0.61) × 10^4^
k_q_ [M^−1^·s^−1^]	(9.48 ± 1.88) × 10^12^	(11.50 ± 1.21) × 10^12^

## Data Availability

Not applicable.
